# De novo transcriptome assembly, functional annotation, and expression profiling of rye (*Secale cereale* L.) hybrids inoculated with ergot (*Claviceps purpurea*)

**DOI:** 10.1038/s41598-020-70406-2

**Published:** 2020-08-10

**Authors:** Khalid Mahmood, Jihad Orabi, Peter Skov Kristensen, Pernille Sarup, Lise Nistrup Jørgensen, Ahmed Jahoor

**Affiliations:** 1 Nordic Seed A/S, Grindsnabevej 25, 8300 Odder, Denmark; 2 Department of Agroecology, Faculty of Science and Technology, Aarhus University, Forsøgsvej 1, Flakkebjerg, 4200 Slagelse, Denmark; 3 Department of Plant Breeding, The Swedish University of Agricultural Sciences, 23053 Alnarp, Sweden

**Keywords:** Agricultural genetics, Gene expression, Plant breeding, Plant sciences, Agroecology

## Abstract

Rye is used as food, feed, and for bioenergy production and remain an essential grain crop for cool temperate zones in marginal soils. Ergot is known to cause severe problems in cross-pollinated rye by contamination of harvested grains. The molecular response of the underlying mechanisms of this disease is still poorly understood due to the complex infection pattern. RNA sequencing can provide astonishing details about the transcriptional landscape, hence we employed a transcriptomic approach to identify genes in the underlying mechanism of ergot infection in rye. In this study, we generated de novo assemblies from twelve biological samples of two rye hybrids with identified contrasting phenotypic responses to ergot infection. The final transcriptome of ergot susceptible (DH372) and moderately ergot resistant (Helltop) hybrids contain 208,690 and 192,116 contigs, respectively. By applying the BUSCO pipeline, we confirmed that these transcriptome assemblies contain more than 90% of gene representation of the available orthologue groups at *Virdiplantae odb10*. We employed a de novo assembled and the draft reference genome of rye to count the differentially expressed genes (DEGs) between the two hybrids with and without inoculation. The gene expression comparisons revealed that 228 genes were linked to ergot infection in both hybrids. The genome ontology enrichment analysis of DEGs associated them with metabolic processes, hydrolase activity, pectinesterase activity, cell wall modification, pollen development and pollen wall assembly. In addition, gene set enrichment analysis of DEGs linked them to cell wall modification and pectinesterase activity. These results suggest that a combination of different pathways, particularly cell wall modification and pectinesterase activity contribute to the underlying mechanism that might lead to resistance against ergot in rye. Our results may pave the way to select genetic material to improve resistance against ergot through better understanding of the mechanism of ergot infection at molecular level. Furthermore, the sequence data and de novo assemblies are valuable as scientific resources for future studies in rye.

## Introduction

Rye (*Secale cereale* L.) is a widely popular cereal crop, especially in northern Europe, due to its high nutritional value and tolerance to unfavorable environmental conditions. Rye remains an important grain crop for cool temperate zones under marginal soil conditions, particularly the ones not suitable for wheat^1^. Rye is used as food, feed and for bioenergy production worldwide^1^. Rye flour is used for making bread and consumed widely in North and Central Europe^2^. The genome of rye consists of seven chromosomes (2n = 14) with a total estimated genome of size of ≈ 8000 Mb^3^. Until recently, compared to sequencing information of other closely related cereals, the least number of genomic DNA and cDNA sequences was found for rye.

Ergot caused by *Claviceps purpurea* is a noxious disease of rye and has been a problematic for rye farmers throughout the history of mankind and agriculture. Ergot is known to cause severe problems in cross-pollinated rye by contamination of harvested grains^4^. Ergot is a serious threat to bread production as milling industry accepts only limited amounts of sclerotia (< 0.05%) from *C. purpurea* in the harvested grains for bread making^4^. The mycotoxins produced by ergot are ubiquitously present and contamination levels exceeding the EU maximum levels can lead to significant economic losses to farmers^5^. In addition, *C. purpurea* represents an interesting biotrophic model system, as no visible signs of plant defense reactions occur in infected plant tissue. Interestingly, during the infection process, the fungus is able to keep the host organism alive for an extended period of time and the fungus is thus classified as true biotroph^6^. *C. purpurea* infects exclusively young unfertilized ovaries. However, the basis of the organ specificity is not clear and until recently no resistance genes were known in cereals. Ergot infection has a complex infection pattern and the molecular response of underlying mechanisms of this disease is still largely unknown, hence gene expression profiling after infection will be useful.

Next Generation sequencing of RNA (RNAseq) can provide astounding details about the transcriptional landscape of an organism. It is the preferred method for cataloguing and quantifying the complete set of transcripts for a specific tissue, developmental stage or physiological condition in response to a specific stress^7^. However, the major challenge of this technology is to assemble a high quality reference transcript set from short read data^8^. RNAseq is a preferred technology to understand morphology^9^, physiology^10^ and microbe-plant interaction^11^. However, there has been limited application of this technology in case of *S. cereal*. There is no universal accepted pipeline to deal with the vast amount of data generated in RNAseq^12,13^. Plant researchers design experiments and adopt different analysis strategies depending on the species being studied and their research goals. Many variations of RNAseq protocols and analyses have been published, making it challenging for researchers to identify best practice for conducting an RNAseq study^12,13^. The challenge starts by dealing with the processing of raw data and then continue with identification of genes in expression profiling and interpretation of the obtained information. The straightforward analysis of RNA sequencing reads is based on an existing reference genome and annotated gene models. However, rye is a non-model plant species and only a draft genome is available^3^. Trimmomatic, which is an efficient tool that can correctly handle paired-end reads was used for the preprocessing of raw data^14^. After preprocessing, reads were assembled using Trinity, which produces good-quality assemblies at single *k*-mer^15^. The Trinity assembler was selected as it has been shown to be better than other de novo assemblers at reconstructing full-length transcriptome^16,17^. The de novo transcriptome assembly in plants is complex due to sequence similarity of transcripts that are isoforms, paralogs and orthologs that may lead to imperfect assemblies in the form of bubbles or extra branches in de Bruijn graphs 18. We employed the CD-HIT tool to refine de novo assemblies by removing the shorter redundant sequences based on sequence similarity^19^. Numerous methods were applied to assess the overall quality, accuracy, contiguity, and completeness of the de novo assembled transcriptomes. The most common is through measuring the proportion of RNA-seq reads used to generate each assembly that map back to the assembled transcriptome. This method is common for RNA-seq analysis, when no reference genome is available. It has the advantages that assemblies are species specific and mapping to transcript assemblies is usually contiguous instead of spliced. The completeness of the de novo assemblies were assessed through Benchmarking Universal Single-Copy Orthologs (BUSCO) that indicate the number of conserved orthologs present in transcriptome^20^. After de novo assembly and quality assessments, we performed functional annotations and determined differentially expressed genes (DEGs). Overall, we presented a stringent workflow in this study for processing of RNAseq data, obtaining de novo assembly, assessment of assembly, functional annotation and differential gene expression from spikes of the non-model plant *S. cereale*.

In this Genomic era, knowledge about the ergot disease has enhanced considerably but the fundamental underlying mechanisms are still poorly understood. Considerable variation in ergot resistance exists among rye germplasms^21–23^. However, little genetic information are available due to the complex unknown infection pattern and huge genome size of rye^15,16^. Hence, an understanding of the underlying molecular mechanisms will assist in breeding of ergot resistant *S. cereale* hybrids. RNA sequencing is a highly efficient method, but false positives still occur due to sensitivity of this technology. Keeping this drawback in mind, we used both a de novo assembled and the draft reference genome of rye to count the differentially expressed genes in a moderately ergot resistant compared to an ergot susceptible hybrid with and without inoculation. When using a draft reference genome, sequence divergence between reads and the rye reference genome may compromise results; nucleotide mismatches are more likely to decrease the number of mapped reads. However, indels are usually better tolerated with gapped alignments^24^. Similarly, in case of de novo assembly a large number of erroneous contigs produced by the assemblers might result in an unreliable estimation. Hence, it remains challenging to analyze the quantification reliability of the assembled contigs generated from de novo assembly. However, utilization of de novo assemblies avoids the mapping issues raised in case of draft genome and captures divergent and novel genes useful for species-specific discovery of new functions. Hence, we performed read quantification based on rye draft genome and de novo assembly generated in this study. These two different datasets are used to support the validity of the method and to DEGs. This analysis method has been shown to produce comparable gene expression profiles in other systems but has not been applied in RNAseq studies of plants^25,26^. Enrichment analysis of gene ontology (GO) terms and gene set enrichment analysis (GSEA) were applied to identify potential pathway responsible to cause ergot infection. Moreover, the behavior and expression profiles of orthologs of identified genes in *Triticum aestivum* were explored. To the best of our knowledge, this is the first attempt with proper experimental considerations to unravel genes involving in ergot infection mechanism in rye hybrid.

## Material methods

### Plant material

**Figure 1:**
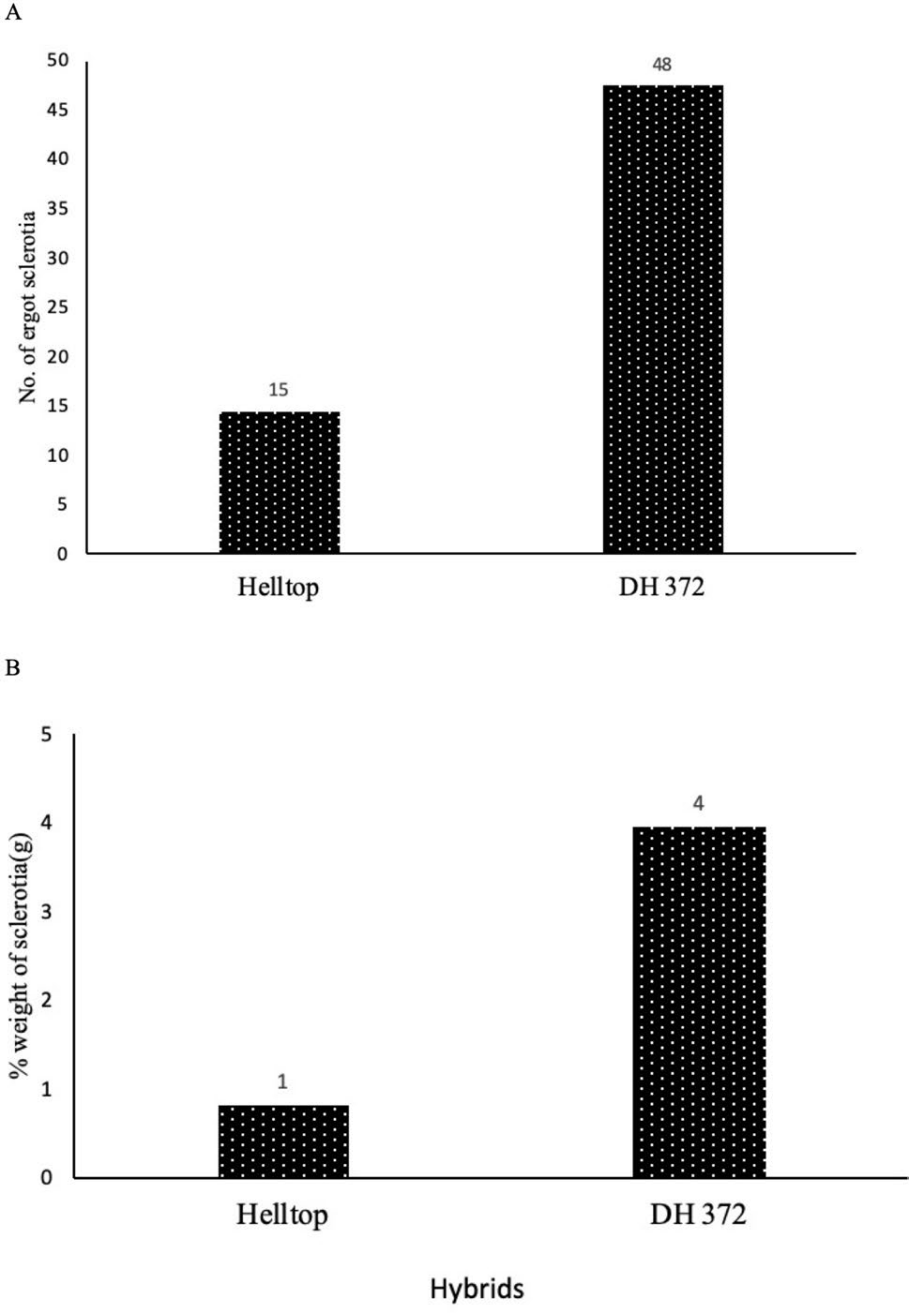
Field trial data of Helltop and DH372 after ergot inoculation. (**A**) Number of ergots per 100 spikes. (**B**) Percentage weight of ergot out of total grain weigh. Data is the average of two years i.e. 2018 and 2019.

In a field experiment, we identified moderately ergot resistant and susceptible hybrids. Both hybrids consist of same genetic components except the restorer component. We presented the data as the average of 2-year i.e. 2018 and 2019 (Fig. 1). In both years, we had two replicates of each hybrid. Each hybrid was cultivated in a plot of two m^2^. A triticale guard on each side of hybrids also cultivated on the plot of two m^2^ to avoid pollination with neighboring hybrid. We measured the number of ergot sclerotia per 100 spikes and percent weight of ergot. The hundred spikes were selected randomly from five different places in group of 20 spikes per place from the plot area of specific hybrid and then total number of ergot sclerotia were counted. The percent weight of ergot sclerotia were determined through weighing the sclerotia in the total grain weight of specific rye hybrid harvested from 1m^2^ area. On average, the frequency of ergot in the ears was 3 times higher in DH372 compared with Helltop (Fig. 1A), whereas percent weight of sclerotia in the grains of DH372 was four-fold higher than Helltop (Fig. 1B). Based on our data, we found out that Helltop was moderately ergot resistant and DH372 was highly susceptible.

### Plant material for transcriptome study

The experiment to obtain plant material for comparative transcriptome was conducted in environment-controlled growth chambers. Seeds of the two hybrids were sown in peat pots (diameter, 15 cm) in greenhouse. After two weeks, plants were placed in cold room for vernalization for 8 weeks. The conditions were maintained at 4 °C, 8/16 h day/night cycle, 120 μE m^−2^ s^-1^ light intensity and 80% humidity. After vernalization, plants were moved into two different growth chambers to avoid cross contamination designated as control or treated. The latter were inoculated with spore solution of *C. purpurea*. Both chambers were set at same growth conditions *i.e*. 16 h light (8,000 lx; 18 °C) and 8 h darkness (15 °C). During growth, nutrients were supplemented once a week during watering. At flowering initiation stage, individual spikes were inoculated with either sterilized water (mock control) or with freshly prepared spore suspension of *C. purpurea*. Spore suspension for inoculation were produced on wheat grains as described by Engelke with slight modifications^27,28^. Briefly, fresh cultures were propagated on Potato Dextrose Agar (PDA, Difco) medium incubated at room temperature. Conidia were produced on autoclaved wheat grains. The inoculated kernels were placed in incubation room at 17–19 °C for 21 days. Conidial suspensions from mycelium growth kernels were harvested in sterile water, filtered through cheesecloth, and concentrations determined with a hemocytometer by microscopy. Spore concentration at 1 × 10^6^ conidia/ml was used for inoculation.

After spray, both treatments were covered with plastic bags to maintain humidity and avoid cross contamination. Spikes were collected in triplicate 5 days post inoculation and immediately frozen in liquid nitrogen and stored at − 80 °C until RNA extraction.

### RNA extraction and sequencing

Total RNA was extracted from 50 mg from each of twelve individual spikes using a CTAB method^29^ with slight modifications. Briefly, spikes were grounded separately using Geno/Grinder (SPEX Sample prep. Stanmore. UK) for 45 s at 1.500 Hz. The fine tissue powder was suspended in 1 ml of pre-heated (65 °C) CTAB extraction and 2% β-mercaptoethanol and mixed carefully. The suspension was incubated in thermomixer at 65 °C for 15 min to permit the complete dissociation of nucleoprotein complexes and centrifuge at 12,000×*g* for 5 min at 4 °C. Later, supernatant was transferred to a new 2 mL tube and added 400 μl of chloroform per 1 mL of CTAB. The tubes were shaken vigorously for 15 s and centrifuged at 12,000×*g* for 10 min at 4 °C. Following centrifugation, aqueous phase was transferred into a new micro-centrifuge tube, and step of centrifugation was repeated. The aqueous phase was collected and precipitated with one third volume of 8 M LiCl and incubated overnight at 4 °C, followed by centrifugation at 16,000 rpm for 30 min at 4 °C. Supernatant was removed and pellet was washed with 200 µL70% ETOH, followed by centrifugation at 13,000 rpm for 10 min at 4 °C. RNA quality was verified using a spectrophotometer (Nanodrop 3,300. Wilmington. USA) and Bioanalyzer (Agilent 2,100. Santa Clara. USA). Samples that showed higher RIN value were treated with DNAse Ambion DNA-free DNase Treatment and Removal kit (Cat #AM1906, California, USA) following the manufacturing protocol (https://www.thermofisher.com/order/catalog/product/AM1906#/AM1906). After DNAse treatment, RNA quality was analyzed again using Bioanalyzer. The mRNA of selected samples was fragmented and transformed to 100 bp short insert strand specific cDNA libraries for sequencing on DNBseq PE100 from BGI (Europe). The raw reads from this library has been deposited in sequence read archive (SRA) with submission ID “PRJNA612415” (https://dataview.ncbi.nlm.nih.gov/object/PRJNA612415?reviewer=r5fg70lms7oandmb7aid7p0mib).

### Assembly normalization and quality assessment

The raw reads of twelve individual assemblies were filtered to remove low quality reads and reads containing adapter sequence before performing the assembly using the software Trimmomatic (version 0.36) with default settings^14^. The raw reads with a Phred score of base quality below 30 containing only “N” and/or length below 80 bp were removed. These were normalized using a kmer size of 20^30^. This step progressively removed high coverage reads from short read data sets and decreased the sampling variation, discarding redundant data, and removing the majority of errors. Furthermore, the assembly procedure was a combination of different methods. First, the Trinity pipeline (version 2.4) was used with a minimum contig length of 200 bp and a minimum kmer covariance of 2. The longest isoform for each gene was selected (perl script by Brian Haas on https://groups.google.com/forum/#!topic/trinityrnaseq-users/cXM1KiJe7dU). After selection of the longest isoform, contigs were merged according to a similarity criterion of 90% in CD-HIT-EST (version 4.6.3)^19^. The contigs were then translated to coding protein sequences using Transdecoder (version 2.0.1) following identification of the longest ORFs^31^. Through merging all reads from individual libraries, we also created a combined dataset belonging to each hybrid. The non-redundant assemblies specific to *S. cereale* were used in downstream functional annotation using Kyoto Encyclopedia of Genes and Genomes (KEGG) and GO enrichment analysis.

The quality of the assemblies was assessed using several methods. First, the assemblies were evaluated against a database of single copy orthologous genes for plants as implemented in BUSCO (version 1.161)^20^. After BUSCO assessment, the processed RNAseq reads were mapped to the combined transcriptome assembly (pRNAseq-2-CTA) to determine percentage of mapped reads, properly paired reads and singleton. Similarly, the processed RNAseq reads were mapped to own transcriptome assembly (pRNAseq-2-OTA). The information obtained in both analysis were used to assess the quality of de novo assembly as described by Zhao et al.^32^.

### Functional annotation KEGG and GO enrichment

The assemblies were aligned against the NCBI non-redundant protein sequence using BLASTX searches database in the OmicsBox program and only the best homologue was reported. GO mapping was performed against the Gene Ontology database implemented in OmicsBox (version 1.2.4)^33^. Sequences that shared similarities with known protein sequences in BLASTX searches with significant similarity (E < 1e^−10^) were identified using the online tool InterProScan 5.0. The OmicBox program was used to assign Gene Ontology (GO) terms to the annotated sequences to predict the functions of the unique sequences with an e-value hit filter set to 1 × 10^−3^, annotation cutoff of 55 and evidence code set to 0.8 for the different categories as implemented in OmicsBox. Furthermore, KEGG analysis was used to identify potential pathways represented in the transcriptomes of DH372 and Helltop^34^. The OmicBox program was used to assign GO terms to the annotated sequences to predict the functions of the unique sequences and encoded translated proteins.

### Identification of differentially expressed genes, annotation and gene ontology

The raw reads were cleaned following the procedure as described in section above and then these clean reads were aligned to the de novo assemblies to count reads. Similarly, these reads were also aligned to the available draft reference genome of rye^3^. Differential gene expression analysis was performed in OmicsBox (Version 1.2.4) using the package EdgeR (Version 3.11) with FDR correction ≤ 0.05, *P* value ≤ 0.01, fold change ≤ 2 or ≥ 2^35^. EdgeR was also used to normalize the expected counts for relative expression and effective library size using the Trimmed Mean of M-values (TMM) normalization method. Genes with at least 1 count per million (CPM) were selected for further differential expression analysis. Differentially expressed genes (DEG) with FDR ≤ 0.05 and log fold change (logFC) of two were extracted for further analysis for gene ontology and enrichment analysis.

### Expression profile and behavior of wheat orthologs

The orthologues of identified genes involved in pectinesterase activity and cell wall modification were extracted from reference genome of *T. aestivum* using sequence similarity searches. All the identified accession numbers of orthologs were analyzed manually and validated through BLAST search using NCBI database (https://blast.ncbi.nlm.nih.gov/Blast.cgi). The behaviors of these genes under various experimental conditions and in different plant parts were explored using the Genevestigator (www.genevestigator.com) at p < 0.05 and fold change > 2. The Genevestigator is a manually curated and well-annotated database of expression profiling from 11 different plant species with more than 26,889 exclusive plant samples (August. 2019).

## Results

### Sequencing, de novo assembly and assessment of assembled transcriptome

**Figure 2:**
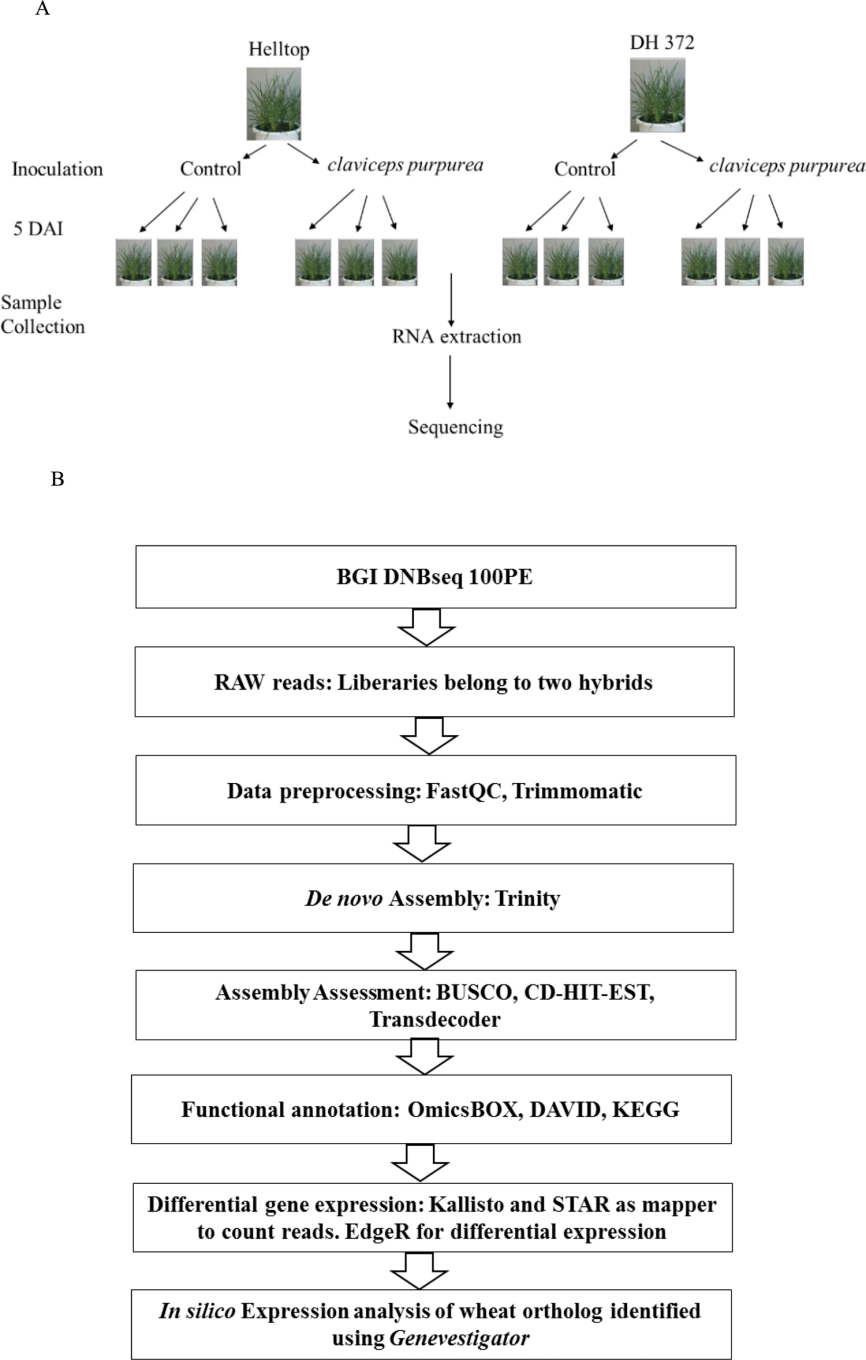
Graphical representation of experimental set up and bioinformatics analysis. (**A**) Experimental set up for RNA extraction from DH372 and Helltop, (**B**) Bioinformatics analysis from raw reads to de novo assembly leading to identification of genes linked to ergot infection.

The clean reads from two *S. cereale* hybrids each containing three independent biological replicates with and without inoculation of *C. purpurea,* respectively (Fig. 2A) were assembled to generate de novo assembled transcriptome (Fig. 2B). The graphical representation of the experimental set up and bioinformatics analysis are shown in Fig. 2. We obtained 18 to 26 × 10^6^ high quality paired-end reads in individual libraries, whereas 137 to 150 × 10^6^ clean reads were assembled into contigs (Table 1). The de novo assembly of these libraries resulted in 82,404 to 98,494 contigs in individual assemblies and 240,689 to 248,337 contigs in pooled assemblies (Table 1). After clustering with CD-HIT-EST, the contigs were assembled into 50,616 to 78,418 transcripts in the individual transcriptomes and 192,116 to 208,690 transcripts of Helltop and DH 372 in the combined transcriptome, respectively. The combined transcriptome assembly of DH372 contains contig lengths between 201 and 16,695 bp and 31,574 contigs are equal or longer than 1000 bp. Similarly, the combined transcriptome assembly of Helltop encompasses contigs between 201 and 14,790 bp, and 25,842 contigs are equal to or longer than 1000 bp. Finally, we identified unigenes in non-redundant combined transcriptome assemblies through blasting against the rye reference genome. This analysis resulted in 18,615 unigenes in combined assembly of DH372, and 18,786 unigenes in Helltop assembly that are used for downstream functional analysis (Table 1).

**Table 1:** Assembly and annotation statistics of de novo assemblies from short read transcriptome data of ergot susceptible DH372 and ergot moderate resistant Helltop of rye hybrids.

Attributes	Ergot susceptible DH372							Ergot resistant Helltop						
	Control			*Claviceps purpurea* inoculated				Control			*Claviceps purpurea* inoculated			
	ID_1A	ID_2A	ID_3A	ID_4A	ID_5A	ID_6A	Combined DH372	ID_10A	ID_11A	ID_12A	ID_13A	ID_14A	ID-15A	Combined Helltop
Clean reads	24,833,832	24,427,226	24,635,234	18,116,390	20,833,628	24,898,586	137,744,896	24,674,052	24,427,654	26,642,152	24,503,442	25,138,406	24,792,620	150,178,326
Assembled bases	2,483,383,200	2,442,722,600	2,463,523,400	1,811,639,000	2,083,362,800	2,489,858,600	13,774,489,600	2,467,405,200	2,442,765,400	2,664,215,200	2,450,344,200	2,513,840,600	2,479,262,000	15,017,832,600
GC %	52.54	52.77	52.42	50.89	50.46	50.23	51.5	52.2	51	50.97	51.26	50.73	51.78	51.32
Transcript number after Trinity assembly	88,023	84,932	89,825	93,016	91,345	89,342	240,689	92,491	90,991	98,494	88,034	82,404	87,345	248,337
Transcript number after CD-HIT-EST	57,143	47,329	50,616	77,315	62,145	61,134	208,690	53,803	61,158	78,418	58,602	53,529	61,232	192,162
Coding sequences (Rye specific)	36,256	31,603	32,609	42,610	31,235	32,342	66,350	34,888	31,731	43,940	35,299	28,529	32,234	57,965
Rye Unigenes	14,978	13,659	13,951	15,347	13,182	13,092	18,458	14,625	13,478	15,316	14,572	9,358	14,765	18,786
Average contig length	723	694	722	737	743	726	766	722	745	869	802	765	863	787
N50	888	834	900	927	908	899	1220	856	875	998		905	723	1235

BUSCO assessment of the quality of transcriptome assemblies revealed that individual transcriptome assemblies have low gene representation (41.4 to 60%), low completeness (34.88 to 53.94%), higher fragmentation (27.21 to 38.84%) and higher missing sequences (11.16 to 26.74%) compared with the combined assemblies. The combined assemblies of DH372 and Helltop possess high percentage of gene representation (91.16 to 91.86%), high completeness (71 to 72%), low percentage of fragmented (7.21 to 7.44%) and very low missing sequences (0.7 to 1.63%) (Table 2). In the assessment using the pRNAseq-2-OTA, percentage of clean reads mapped back to own transcriptome is in range of 82.6 to 89.1% in case of individual assemblies and 91.9 to 92.3% of clean reads mapped to their respective combined assemblies (Table 2). Similarly, in pRNAseq-2-CTA, clean reads mapped to individual assemblies were in the range of 93.8 to 96.2%, whereas clean reads mapped to the respective combined assemblies were in the range of 96.1–96.3%. In the assessments of pRNAseq-2-CTA, we observed higher percentage of mapped and properly paired reads compared to pRNAseq-2-OTA, whereas the % of singletons are low. After all the redundancy reduction steps and obtaining good quality of assembly specific to *S. cereale*, we decided to dissect the annotation of these assembled sequences in the respective combined assemblies.

**Table 2:** Assessment of the assemblies using Benchmarking Universal Single-Copy Orthologs (BUSCO) and read mapping.

Attributes	Ergot Susceptible DH372							Ergot Moderate Resistant Helltop						
	Control			*Claviceps purpurea* inoculated				Control			*Claviceps purpurea* inoculated			
	ID_1A	ID_2A	ID_3A	ID_4A	ID_5A	ID_6A	Combined DH372	ID_10	ID_11	ID_12	ID_13	ID_14	ID-15	Combined Helltop
**BUSCO (%)**														
Gene Representation	60	44.8	49.07	49.3	41.4	43.34	91.16	53.49	39.07	48.14	57.91	46.05	49.23	91.86
Complete single copy	53.95	40.7	45.81	43.26	36.98	38.12	71	46.74	34.88	44.42	52.79	38.84	43.78	72
Duplicated	6.05	4.19	3.26	6.05	4.42	5.22	20.16	6.74	4.19	3.72	5.12	7.21	5.54	19.86
Fragmented	28.84	30.93	31.16	34.65	35.35	37.45	7.21	30.7	38.84	33.49	28.37	27.21	32.12	7.44
Missing	11.16	24.19	19.77	16.05	23.26	19.21	1.63	15.81	22.09	18.37	13.72	26.74	18.65	0.7
**pRNAseq-2-OTA**														
% mapped	84.1	86.2	82.6	81.6	89.1	84.5	91.9	86.9	88.3	82.8	83.6	86.9	84.2	92.3
% properly paired	77.9	78.5	77.6	75.9	80.1	78.5	82.9	78.2	81.4	74.2	77.3	78.1	76.7	81.3
% singletons	3.9	3.3	3.4	3.7	4.3	3.8	3.3	3.2	3.4	3.5	3.1	3.5	3.2	2.2
**pRNAseq-2-CTA**														
% mapped	95.5	96.3	96.0	95.4	96.2	94.5	96.1	93.8	95.1	94.6	94.2	94.1	94.2	96.3
% properly paired	86.2	85.3	88.2	87.2	88.4	87.5	89.1	86.9	88.1	87.3	86.9	86	87.1	89.3
% singletons	1.6	1.7	1.0	1.2	1.3	1.4	1.0	1.3	1.3	1.4	1.3	1.1	1.6	1.0

pRNAseq-2-OTA: the processed RNAseq reads were mapped to own transcriptome assembly.

pRNAseq-2-CTA: the processed RNAseq reads were mapped to combined transcriptome assembly.

### Functional annotation of non-redundant combined de novo assemblies of DH372 and Helltop

In the combined transcriptome assemblies, most sequences got hit to *Tritucum asestivum* (87%, 16,378 unigenes in DH372 and 16,644 in Helltop), followed by *Hordeum vulgare* (5%, 1030 in DH372 and 1,034 in Helltop) and *Brachypodium distachyon.* The top 10 organisms all belong to the *Poaceae* family and 92% of the contigs got a top hit to *Triticeae* (Supplementary Figs. 1A and 1B). Both assemblies had a similar percentage of hits to *Triticum aestivum* and *Hordeum vulgare*. However, a difference was observed in case of *Sorghum bicolor* and *Eragrostis curvula*, where the share of contigs in the assembly of Helltop are higher than in DH372 (Supplementary Fig. 1C).

**Figure 3:**
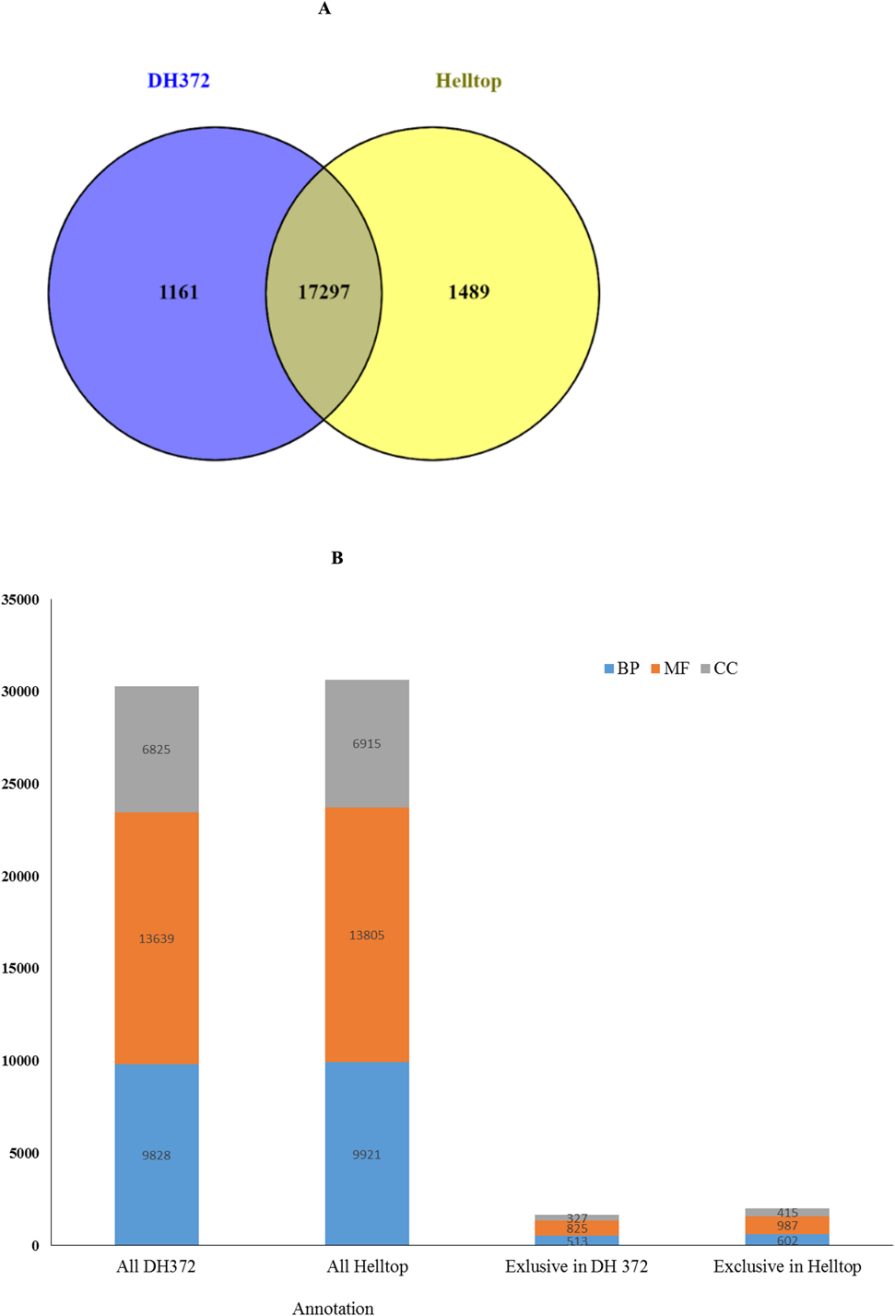
Distribution of the Unigenes sequences (**A**) and gene ontology (**B**) in the assembly of DH372 and Helltop. (**A**) Count of unigenes in the transcriptome assemblies of Helltop and DH372 (Helltop = unigenes identified within transcriptome assembly of Helltop; DH372 = unigenes identified within transcriptome assembly of DH372). (**B**) The number of GO terms linked to unigenes identified in the transcriptome assemblies of Helltop, DH372 and unigenes exlusively found in either of transcriptome assembly.

**Figure 4:**
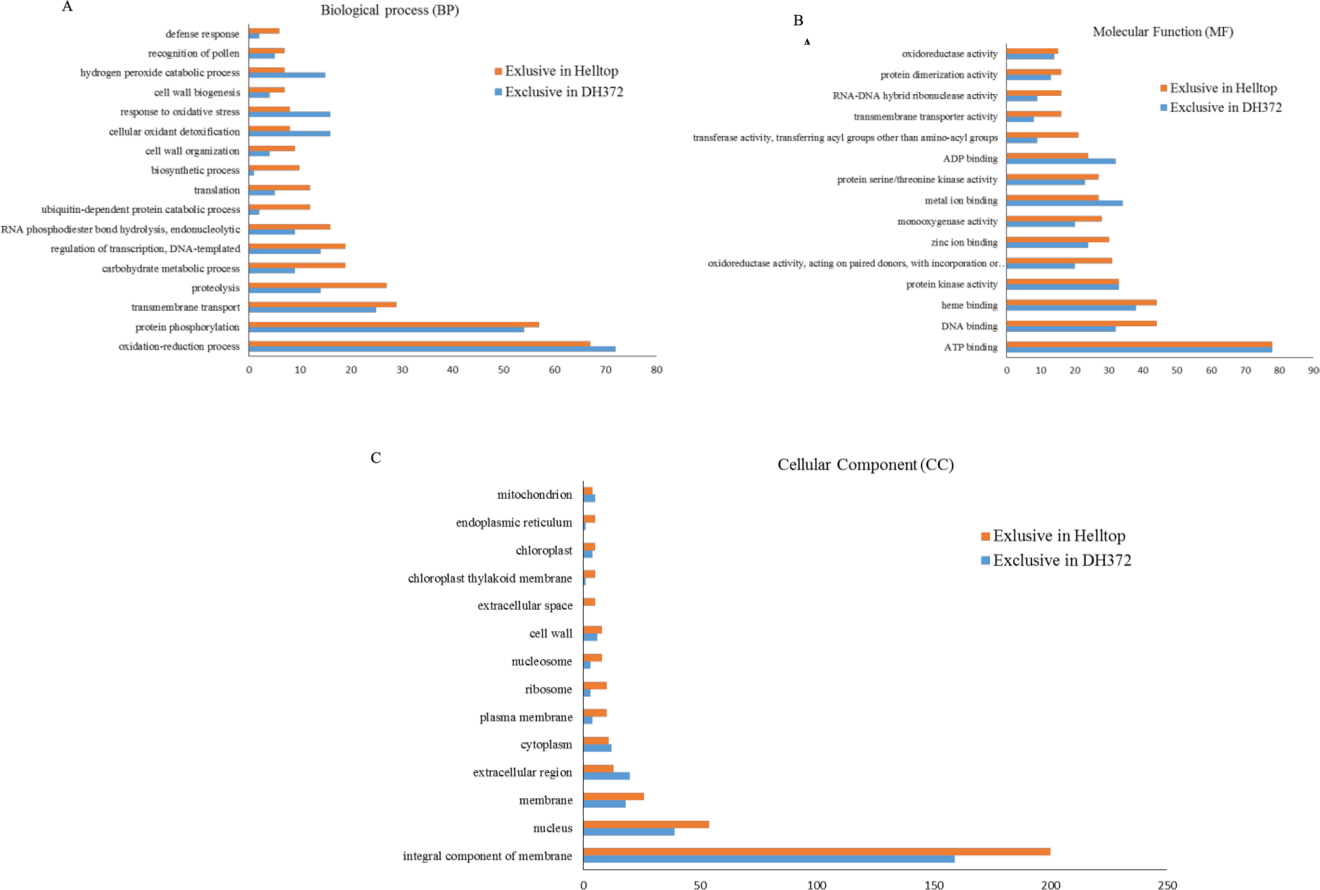
Distribution of Gene ontology terms in genes exclusively present in the assemblies of DH372 and Helltop. (**A**) Biological process, (**B**) Cellular component, (**C**) Molecular function.

In the GO analysis, a total of 9282, 13639 and 6825 unigenes of DH372 were assigned to biological processes (BP), molecular functions (MF) and cellular components (CC) GO categories, respectively. Similarly, unigenes of Helltop were assigned to 9921, 13,805 and 6915 of BP, MF and CC categories, respectively (Fig. 3A). Top categories of BP, MF and CC of the hybrids were found very similar in both assemblies (Supplementary Fig. 2). In both assemblies, highest number of unigenes in BP was associated with oxidation reduction process (9%), protein phosphorylation (8%) and regulation of transcription (5%). In MF, unigenes of both assemblies were predominantly assigned to ATP binding (10%), DNA binding (4%), metal ion binding (4%) and protein kinase activity (3%). Similarly, in the CC category, unigenes were abundant as integral component of membranes (42%), nucleus (13%) and cytoplasm (3%) (Supplementary Fig. 2). Overall, comparison of functional annotations between the assemblies of Helltop and DH372 showed that these are rather similar in their GO annotations. As both hybrids have differential responses towards ergot, we decided to identify unigenes exclusively found in each hybrid and linked them to various GO terms. We identified 1161 unigenes exclusive to DH372 and 1489 unigenes found only in Helltop de novo assembly (Fig. 3B). In the top GO terms, difference in BP, MF and CC of exclusive genes of DH372 and Helltop were more pronounced compared to all unigenes in the both hybrid assemblies. In the BP category, there was a higher number of Helltop genes was associated to proteolysis, biosynthetic process, ubiquitin dependent protein catabolic process, cell wall organization, defense response and recognition of pollen, whereas genes associated with oxidation reduction process, cellular oxidant detoxification, oxidative stress and hydrogen peroxide catabolic process was predominant in DH372 (Fig. 4). In MF category, higher number of genes found in Helltop were related to DNA binding, oxido-reductase activity, transferase and mono-oxegenase activity, transporter activity and ribonuclease activity. Similarly, in CC category, Helltop had higher number of genes linked to integral component of membrane, localized in nucleus, plasma membrane, extra cellular space and endoplasmic reticulum (Fig. 4).

**Figure 5:**
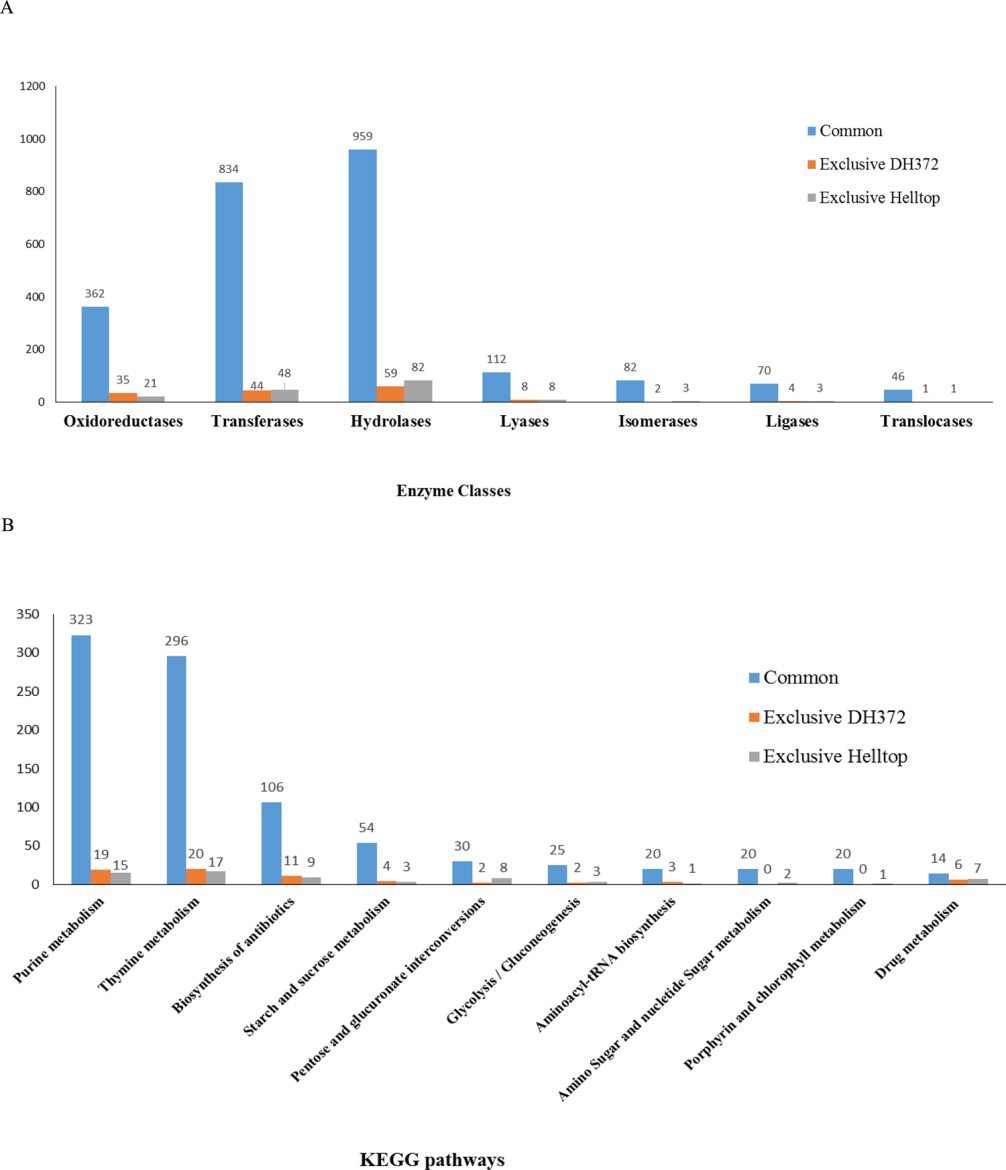
Distribution of unigenes sequences of DH372 and Helltop to enzyme classes and KEGG (Kyoto Encyclopedia of Genes and Genome) pathways. The major classes of enzymes (**A**) and top 10 most highly represented KEGG pathways (**B**) are shown. Analysis was performed using the OmicsBox and the KEGG database, and unigenes were assigned to biological pathways in the KEGG pathway (https://www.genome.jp/kegg/).

DH372 and Helltop have 2613 and 2634 unigenes sequences that have enzyme codes and these enzyme coding sequences represented 14% of the respective assemblies. The highest number of these genes were associated with hydrolases followed by transferases and oxidoreductases in the both assemblies (Fig. 5). Kyoto Encyclopedia of Genes and Genomes (KEGG) analysis was used to identify potential pathways most represented in assembly of Helltop and DH372. The top ten KEGG pathways based on sequences number were compared between assemblies of Helltop and DH372. All assemblies have similar pathways in their top ten such as important metabolisms pathways (purine, thymine, starch and sucrose, Porphyrin and chlorophyll) and biosynthesis pathways (biosynthesis of antibiotics, gluconeogenesis, aminoacyl tRNA biosynthesis), pentose and glucoronate interconversions and drug metabolism. These most represented pathways are important basic pathways necessary for cell life and therefore naturally found in our assemblies. Purine metabolism was the pathway with the most sequences in each individual assembly with 348 and 338 unigenes in DH372 and Helltop, respectively. In case of unigenes exclusively assembled in each hybrid, thymine metabolism was the pathway with more genes. The major differences were observed in drug metabolism and pentose and glucoronate interconversions pathways. In the pentose and glucoronate interconversions, 8 unigenes were exclusively found in Helltop compared to 2 in DH372 (Fig. 5B). Similarly, in drug metabolism pathway, 7 unigenes were exclusively found in Helltop and 6 in DH372 (Fig. 5B).

### Differential expression and enrichments analysis

**Figure 6:**
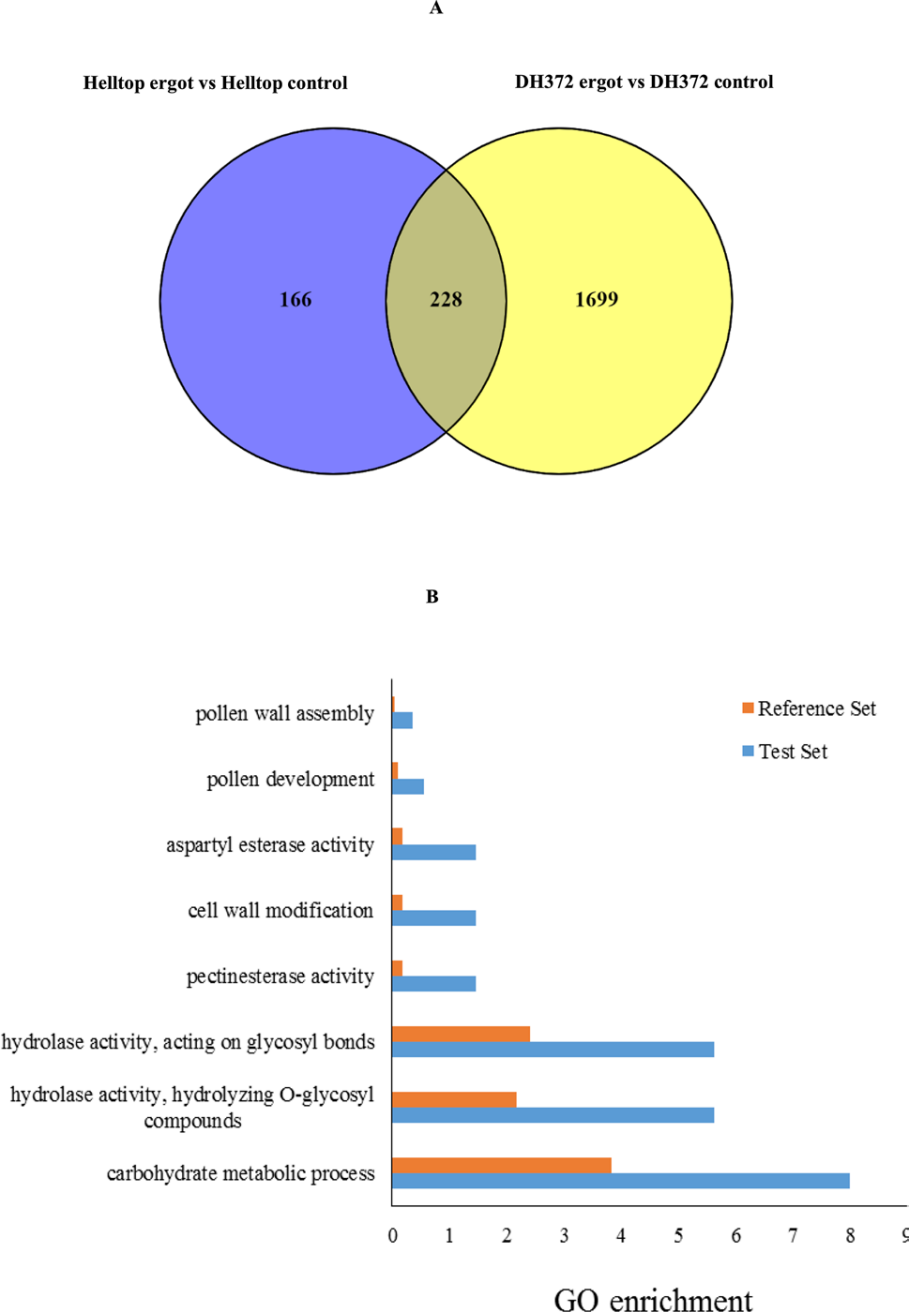
Comparison and Gene Ontology enrichment analysis of differentially expressed genes. (**A**) Counts of DEGs identified in Helltop and DH372 after ergot infection (Helltop = DEGs identified within Helltop; DH372 = DEGs identified within DH372 after ergot infection (**B**) Significant gene enriched terms found in DEGs identified in both hybrids after ergot infection.

**Figure 7:**
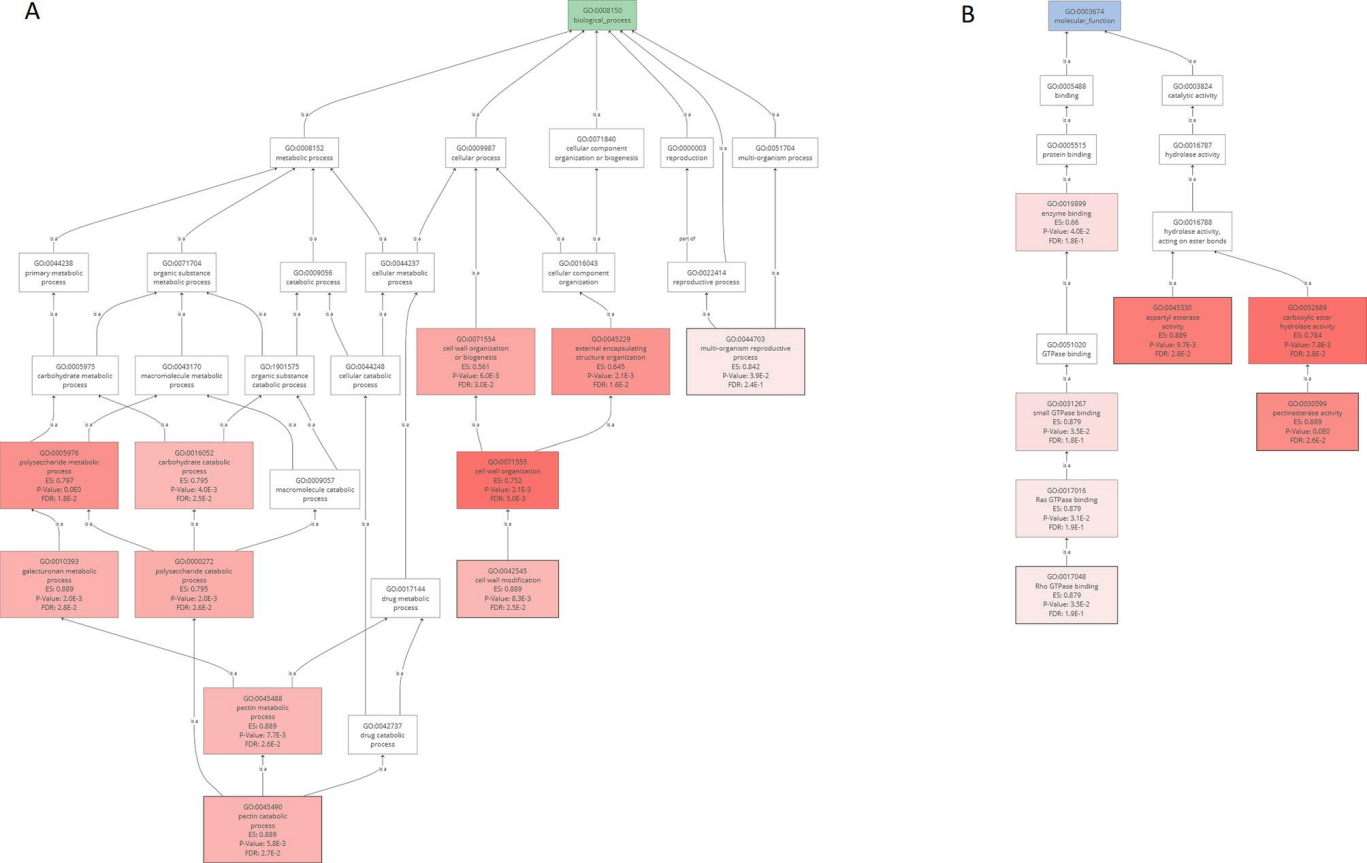
Gene Set Enrichment Analysis (GSEA) of DEGs to identify pathways in Biological processes (**A**) and Molecular functions (MF). Pectin catabolic process and cell wall modification were significant pathway in GESA in biological process and pectinesterase activity and Rho GTPase binding were significant pathways in molecular function.

Reads were counted by mapping to de novo assembled transcriptomes and the rye reference genome for the identification of differentially expressed genes (DEGs) between the two hybrids. The number of DEGs identified in the different comparisons can be found in Table 3. However, here onwards, we will describe only the genes that were common in both genomes to support the validity and reliability of our analysis. A higher number of DEGs were identified when comparison was made between control and inoculated samples of hybrids than the comparison between controls of the both hybrids or between infected plants of both hybrids (Table 3). In case of Helltop after ergot infection, in total 394 DEGs were identified, where 324 DEGs were up-regulated and 70 DEGs were down-regulated. Interestingly, a large number of DEGs were identified in DH372 (Table 3). Furthermore, the comparison of identified DEGs in Helltop and DH372 after ergot infection were presented in Venn diagram (Fig. 6). In this comparison, a far greater number of DEGs were specific to the ergot-susceptible DH372 than to Helltop, consistent with increased activity of ergot-activated transcriptional regulators in DH372 (Fig. 6). The genes that were differentially expressed in both hybrids after ergot infection were used for gene enrichment analysis. We identified the significantly enriched GO terms of these DEGs that were significantly associated to carbohydrate metabolic processes, hydrolase activity, pectinesterase activity, cell wall modification, pollen development and pollen wall assembly (Fig. 6). After GO terms enrichment analysis, we decided to use gene set enrichment analysis (GSEA) to identify the most significant pathways. The GSEA analysis identified pectin catabolic process and cell wall modification in biological process category, whereas pectinesterase activity and Rho GTPase binding were significant pathways in molecular function (Fig. 7). After identifying these pathways, we decided to dissect the genes involved in these pathways in detail in both hybrids after ergot infection and the list of genes are presented in Table 4. In case of cell wall modification and pectinesterase activity, in total 12 and 8 genes are differentially upregulated in response to ergot infection in both hybrids, respectively (Table 4). Among those genes, six genes named “XLOC_059237” “XLOC_1003867”, “XLOC_118963”, “XLOC_1220465”, “XLOC_1387037” and “XLOC_386424” are present in cell wall modification and pectinesterase pathways (Table 4). The COBRA-like 10 (XLOC_1432429) and probable pectinesterase inhibitor 21 (XLOC_118963) involved in cell wall modification were topmost differentially expressed with 2465- and 2261-fold changes, respectively. Interestingly, pectinesterase inhibitor 21 was also found in pectinesterase activity pathways. These two genes (XLOC_1432429, XLOC_118963) were also listed in top 30 differentially expressed genes in both hybrids after ergot infection (Supplementary Table 1). In cell wall modification pathway, three DEGs named “XLOC_1343481”, “XLOC_1343482” and “XLOC_145869” were found to be involved in Polygalacturonase. These results prompted us to dissect the orthologs of these genes in *T. aestivum* in order to identify their expression and behavior under various conditions using publicly available transcriptome data.

**Table 3:** Differentially expressed genes (DEGs) in both hybrids in various comparisons.

Treatment Comparison	Total DEG in reference /de novo	Common in both datasets	Down-regulated genes	Up-regulated genes
Helltop control versus DH372 control	116/189	116	44	72
Helltop ergot versus DH372 ergot	37/158	36	12	24
Helltop Ergot versus Helltop Control	494/1133	394	70	324
DH 372 ergot versus DH372 control	3633/1930	1927	824	1103

**Table 4:** List of genes in Pectinesterase activity and cell wall modification pathways with their expression values. These two pathways identified through Gene set enrichment analysis (GSEA).

Name	Blast Description	Length	Tags	FC	logFC	logCPM	*P* Value	FDR	Wheat orthologs
**Pectinesterase activity**									
XLOC_059237	unnamed protein product	630	[UP]	1109	10	5	7.82E−04	0.043604	TraesCS5B02G458900
XLOC_1003867	pectinesterase-like	3824	[UP]	1040	10	7	1.29E−04	0.021255	TraesCS5A02G216500
XLOC_118963	probable pectinesterase/pectinesterase inhibitor 21	2829	[UP]	2261	11	8	9.12E−05	0.018767	TraesCS2D02G091800
XLOC_1220465	unnamed protein product	2318	[UP]	484	9	4	6.69E−04	0.033665	
XLOC_1387037	unnamed protein product	2017	[UP]	529	9	4	0.001595	0.04525	TraesCS5A02G086800
XLOC_146138	probable pectinesterase 53 isoform X2	3241	[UP]	8	3	3	2.90E−04	0.026195	TraesCS2D02G099900
XLOC_149812	unnamed protein product	3246	[UP]	6	2	5	0.0018072	0.047246	TraesCS6A02G351000
XLOC_386424	unnamed protein product	2991	[UP]	56	6	3	0.0010457	0.037915	TraesCS1B02G274500
**Cell wall modification**									
XLOC_059237	unnamed protein product	630	[UP]	1109	10	5	7.82E−04	0.03436	TraesCS5B02G458900
XLOC_1003867	pectinesterase-like	3824	[UP]	1040	10	7	1.29E−04	0.021255	TraesCS5A02G216500
XLOC_1018540	cellulose synthase-like protein D3	6970	[UP]	1600	11	7	7.61E−04	0.034291	TraesCS2B02G144900
XLOC_109229	unnamed protein product	2858	[UP]	200	8	1	8.29E−04	0.035588	TraesCS5D02G481800
XLOC_118963	probable pectinesterase/pectinesterase inhibitor 21	2829	[UP]	2261	11	8	9.12E−05	0.018767	TraesCS2D02G091800
XLOC_1220465	unnamed protein product	2318	[UP]	484	9	4	6.69E−04	0.033665	TraesCS3B02G598100
XLOC_1343481	Polygalacturonase	1257	[UP]	88	6	1	1.56E−04	0.022086	TraesCS5B02G468900
XLOC_1343482	Polygalacturonase	2823	[UP]	124	7	1	5.03E−04	0.031517	TraesCS5A02G459000
XLOC_1387037	unnamed protein product	2017	[UP]	529	9	4	0.001595	0.04525	TraesCS5A02G086800
XLOC_1432429	COBRA-like protein 10	4108	[UP]	2465	11	8	9.29E−05	0.018767	TraesCS7D02G136500
XLOC_145869	Exopolygalacturonase	1273	[UP]	1132	10	7	6.95E−04	0.034064	TraesCS6A02G159000
XLOC_386424	unnamed protein product	2991	[UP]	56	6	3	0.0010457	0.037915	TraesCS1B02G274500

*FC* fold changes, *CPM* count per million.

### Expression profile and behavior of wheat orthologs

The majority of wheat orthologs have very similar expression pattern with higher expression during flower initiation stage (Supplementary Fig. 3A). In the quest to understand the function of these genes, we found out that majority of the genes in wheat were not highly expressed under biotic stress category. Orthologues of COBRA-like 10 (TraesCS7D02G136500), probable pectinesterase inhibitor 21 (TraesCS2D02G091800) and Polygalacturonase (TraesCS5B02G468900, TraesCS5A02G459000 and TraesCS1B02G274500) did not exhibit differential expression under various biotic stresses. Notably, orthologues of “XLOC_386424” (TraesCS1B02G274500) had significant up-regulation under various biotic stress (Supplementary Fig. 3B) and orthologs of “XLOC_149812” (TraesCS6A02G351000) exhibit down-regulation after *F. graminearum* infection.

## Discussion

Ergot infections of rye is a severe problem of food security. Although several studies have investigated the infection mechanisms by which *C. purpurea* penetrate spikes, not much is known about the molecular mechanisms of the infection. One obvious reason is the lack of sequence resources for rye during ergot infection, which impose serious limitations to studies the molecular mechanism. In this study, we generated transcriptomic resources, and created two de novo assemblies of rye hybrids with known differential responses to ergot infection. These high-quality transcriptome assemblies may constitute an important step forward for understanding of this highly complex disease and complements the resources recently made available for other fungal diseases in *Triticeae*^36–38^. In a previous transcriptome study, de novo assembly had 115,400 contigs arising from five rye inbreeding lines using Roche/454 GS FLX technology^39^. However, no functional annotation of the aforementioned transcriptome was available for comparison. In this study, we had used Illumina technology to develop transcriptome assemblies of DH372 (ergot susceptible) and Helltop (ergot moderate resistant) during ergot infection and provided annotations (Supplementary Fig. 2 and Fig. 3). Moreover, we provided a comprehensive transcriptome analysis pipeline to deal with vast amount of data due to huge genome size of rye that suited well to decipher molecular mechanism during ergot infection. We employed an assembler, which produces good quality assembly at single k-mer. Numerous methods were applied to assess the overall quality, accuracy, contiguity, and completeness of a de novo assembled transcriptome. BUSCO assessments revealed that the combined transcriptome assemblies have good representation and can serve purpose of perfect de novo assembled transcriptome of rye spikes (Table 2). However, different levels of BUSCO completeness were observed when comparing different individuals of the same hybrids. Thus, indicating that hybrid specific transcripts may account for different coverages in various individual transcriptome assemblies. The quality assessments using the pRNAseq-2-CTA also revealed that the individual assemblies are well represented in the respective combined assembly of DH372 and Helltop. Nonetheless, BUSCO completeness observed in our de novo assemblies were comparable to the completeness of other cereals transcriptomes^40,41^. In crux, all three assessment methods (BUSCO, pRNAseq-2-OTA and pRNAseq-2-CTA) indicate that de novo assembled transcriptomes are of very high quality and will be very useful addition to the molecular toolbox of rye.

Lack of a well annotated rye reference genome and the differential response of hybrids towards ergot prompted us to perform functional annotations on the combined transcriptome assemblies of DH372 and Helltop. In the combined transcriptome assemblies, majority of contigs got top hit to *Triticeae,* which is in line with previous high quality de novo assemblies of wheat, barley and other cereals^42–45^. We also observed that a number of contigs in the de novo assembly of Helltop that got hit to *Sorghum bicolor* and *Eragrostis curvula*. This led us to do GO of both assemblies for the unigenes exclusive to each hybrid. Pronounced differences were observed as in case of Helltop genes associated with proteolysis, biosynthetic process, ubiquitin dependent protein catabolic process, cell wall organization, defense response and recognition of pollen, whereas genes exclusive to DH372 associated with oxidation reduction process, cellular oxidant detoxification, oxidative stress and hydrogen peroxide catabolic process (Fig. 4). Similarly, we observed major differences in drug metabolism pathway and pentose and glucoronate interconversions pathways (Fig. 5). Drug metabolism constitutes genes that belonging to the family of Cytochrome P450s, GSTs, glycosyl transferases and ABC transporters. These genes are known to play a role in plant-pathogen interactions in various plant species. Hence, this is in agreement with the notion that plant defense depends on metabolism of pathogenesis related proteins at pathogen contact site^46–49^. Another predominant pathway was pentose and glucoronate interconversions and this pathway is directly related to the target prediction of UDP-glucuronosyltransferase that can increase the strength of cell walls eventually leading to resistance to pathogens^50^. The enrichment analysis of DEGs revealed that DEGs were associated with carbohydrate metabolic processes, hydrolase activity, pectinesterase activity, cell wall modification, pollen development and pollen wall assembly. Similarly, gene set enrichment analysis (GSEA) lead us to identify cell wall modification and pectinesterase activity as candidate pathways for ergot resistance. These results are very interesting and in line with earlier findings that inhibition of pectin may be a possible route to control ergot in cereals^51^. Pectinesterase activity is known to enhance resistance against different pathogens in pepper^52^, wheat^53^, *Arabidopsis thaliana*^54,55^ , cotton^56^ and pearl millet^57^. Therefore, our transcriptome analysis is very useful to formulate hypothesis that can bring further understanding of underlying genetics of ergot resistance. The identified pathways in our transcriptome analysis are interlinked with each other and hence provide a starting point to explore the genes that cause resistance. In our analysis, we found six genes that are common between two pathways (Pectinesterase activity and cell wall modification). In earlier studies, polygalacturonase is known as a pathogenicity factor in the *C. purpurea*/rye interaction^58^. In cell wall modification pathway, three genes were differentially expressed (Table 4). The esterification through pectinesterase activity affect the cell wall structure leading to cell wall modification and ultimately resistance against pathogens^59^. Cell wall-associated plant defense is critical in basal resistance against fungal pathogens^60^. Plant cell walls are the first essential component of defense and is believed to be a critical factor in plant response to fungal infection by providing a physical impediment between pathogens and the internal contents of the plant cells^61,62^. Based on our transcriptome results, we assumed that rye plants initiate several mechanisms in response to ergot infection and findings of this study provide a good basis to design future approaches to unravel those mechanism and gain a deeper understanding.

Although RNA sequencing is a highly efficient method, false positives still occur because of the sensitivity of this technology. Hence, we had used reference as well as de novo assembled transcriptome datasets to count the DEGs and discussed only those that were found in both (Table 3). The majority of RNAseq experiments involved estimating transcript expression level via read counts, this results in several specific challenges that must be addressed. The abundance of transcripts due to huge genome size of rye makes it likely that transcripts belonging to parts of different genes will assemble, making the measurement of a gene’s transcript counts prone to large errors. Similarly, in case of using only a draft genome, many short reads, particularly those from Illumina-based RNA-seq experiments, are likely to align to identical sequence fragments at several loci and thus may be discarded from transcript counts affecting the estimated expression level for a given gene. The current study utilizes a draft reference genome as well as de novo assembled transcriptome for quantification of gene expression and we obtained comparable expression profile using both approaches. Hence, we believe that the results obtained using this approach are more reliable than using only a draft genome or a de novo assembled transcriptome. Therefore, we assume that the pipeline devised for this RNA-seq experiment in this study for rye are applicable and valuable to most plant RNA-seq experiments having huge genome size. However, we cannot rule out false positive completely as problems of transcriptional responses not always happening at the exact time they are measured or being highly tissue specific leading to "averaging" when bulk tissues are sampled. In future, this issue can be addressed through single cell transcriptome studies at multiple time points after ergot infection.

Among the genes in enriched pathways, The COBRA-like 10 (XLOC_1432429) was one of the top most expressed gene and known to mediate directional growth of pollen tubes in *Arabidopsis thaliana*^63^. It is common notion that *C.* *purpurea* pathogen cause fungal infection through mimicking pollen-tube growth^64,65^. Knockout of the COBRA-like 10 gene caused gametophytic male sterility^63^. Based on available information regarding COBRA-like 10, we can speculate that ergot conidia might not be able to infect the stigmas of flowers in presence of higher expression of this gene. However, at this point its mere speculation and it will be an interesting area to explore further, which might lead to discovery of underlying mechanism of infection and ergot resistance in rye. Moreover, pollen development and pollen wall assembly are significantly enriched terms in our GO term enrichment analysis. The infection process of *C. purpurea* is tissue specific as only flowers of blossoming rye spikes are infected. Probably, it requires specific recognition of the stigmatic surface. However, experimental proof of which specific gene or genes are missing. Early stages of infection show striking parallels to the plant fertilization process, and especially growth of pollen tubes in the pistil tissue^6^. The fungus evidently mimics pollen tube growth and might use specific signal components of the pollen–stigma interaction to avoid recognition by the plant^6^. Nevertheless, all these facts point out a potential role of efficient communication between female tissues and growing pollen tubes during ergot infection. Consequently, candidate genes identified in this study could be valuable assets for future studies.

The expression profiling of wheat orthologues of these genes revealed that they were highly expressed at flower initiation stage. This is again pointing towards significance of flower specificity in ergot infection and a probable role of these genes in ergot resistance during flower development stage. However, under various biotic stresses, majority of these genes remains unaffected and did not exhibit significant differential expression in wheat. This might be due to specific involvement of these genes during ergot infection and resistance in rye but not in wheat. Wheat is a self-fertile plant and flowers are closed, which reduce the risk for ergot infection. Wheat might have different mechanism of ergot infection and resistance due to specific self-pollination strategy and therefor have a different set of candidate genes than those found in rye in this study. There is also lack of expression data during ergot infection in wheat and which could be one reason for non-significant expression in publicly available transcriptome data of wheat. Focusing on gene families whose functional annotation is relevant to the mechanism of ergot infection and phenotype, we dissect their differential expression patterns and identify candidates for the genetic basis of the observed differences. Overall in this study, we provided a strong evidence of involvement of specific set of genes in ergot infection mechanism. We cannot say for certain that these DEGs are related to ergot resistance. However, this group of genes remain interesting candidate genes for future studies such as CRISPR-Cas to validate their function in ergot resistance. Functional annotations of two de novo assemblies along with enrichments analysis of DEGs emphasize the role of cell wall and flower morphology to evolve resistance against ergot in cross pollinated rye.

## Conclusion

Our transcriptomic exploration demonstrates, how ergot influence transcript expression in two hybrids, which has allowed us to identify and annotate transcripts associated with ergot infection. The resource generated in this study will help to lay the foundation to understand the precise nature and origin of ergot infection in rye. This is an indispensable pre-requisite for the development of gene-based strategies that can exploit untapped genetic diversity in breeding materials and ex situ gene banks to improve resistance in rye hybrids. The identification of pathways and set of candidate genes is an important forward step toward a better understanding of the complex mechanism that can be utilized to devise novel strategies of ergot management in the future. In crux, availability of additional transcriptome sets from rye hybrids under specific conditions as presented here will serve as valuable resources for rye breeders and to the research community for further functional and comparative genomics studies.

## Supplementary information

Supplementary information.
