# Supplementary material for: De novo transcriptome assembly, functional annotation, and expression profiling of rye (Secale cereale L.) hybrids inoculated with ergot (Claviceps purpurea)
Source: Sci Rep. 2020 Aug 10;10:13475. doi: 10.1038/s41598-020-70406-2 (PMC7417550; doi:10.1038/s41598-020-70406-2)
Supplement: Supplementary file 1 — Supplementary information. [file 41598_2020_70406_MOESM1_ESM.docx]

**Supplementary information**

***De novo* transcriptome assembly, functional annotation, and expression profiling of rye (*Secale cereale* L.) hybrids inoculated with ergot (*Claviceps purpurea*)**

Khalid Mahmood* ^1,2^, Jihad Orabi ^1^, Peter Skov Kristensen^1^, Pernille Sarup ^1^, Lise Nistrup Jørgensen^2^, and Ahmed Jahoor ^1,3^

**Affiliations:**

^1^Nordic Seed A/S, Grindsnabevej 25, Odder, 8300, Denmark

^2^Department of Agroecology, Faculty of Technology, Aarhus University, Forsøgsvej 1, Flakkebjerg, DK-4200, Slagelse Denmark

^3^ Department of Plant Breeding, The Swedish University of Agricultural Sciences, 23053 Alnarp, Sweden

***Corresponding author:** Khalid Mahmood

Nordic Seed A/S and Department of Agroecology, Faculty of Technology, Aarhus University, Denmark

Email: [khalid.mahmood@agro.au.dk](mailto:khalid.mahmood@agro.au.dk)

Phone: +45-42783010

**B**

**A**


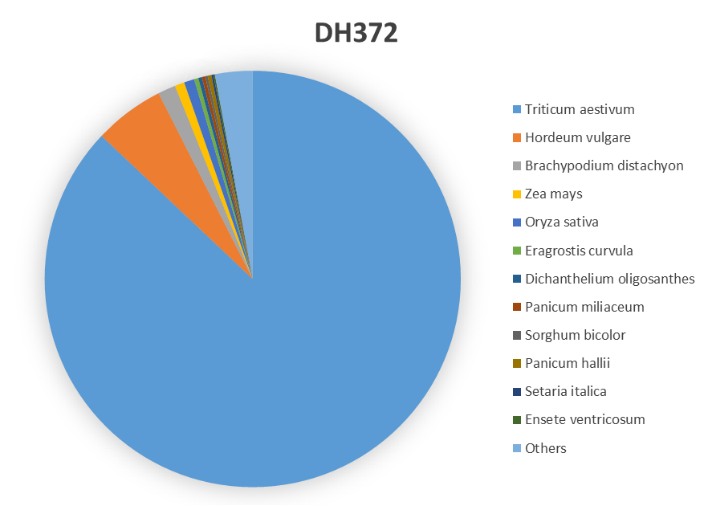

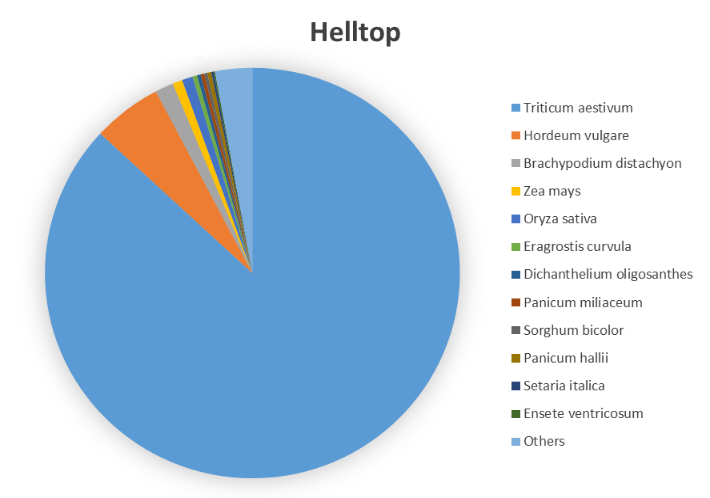


**C**


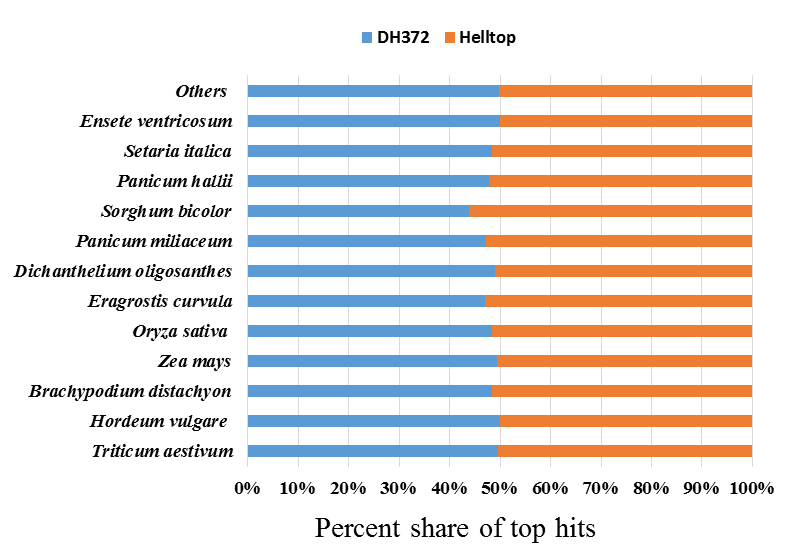


### Supplementary Fig 1: Species distribution of the Unigenes sequences of DH372 and Helltop to the top BLAST hits. A) Top hits of DH372 B) Top hits of Helltop C) Percent share of top hits species distribution


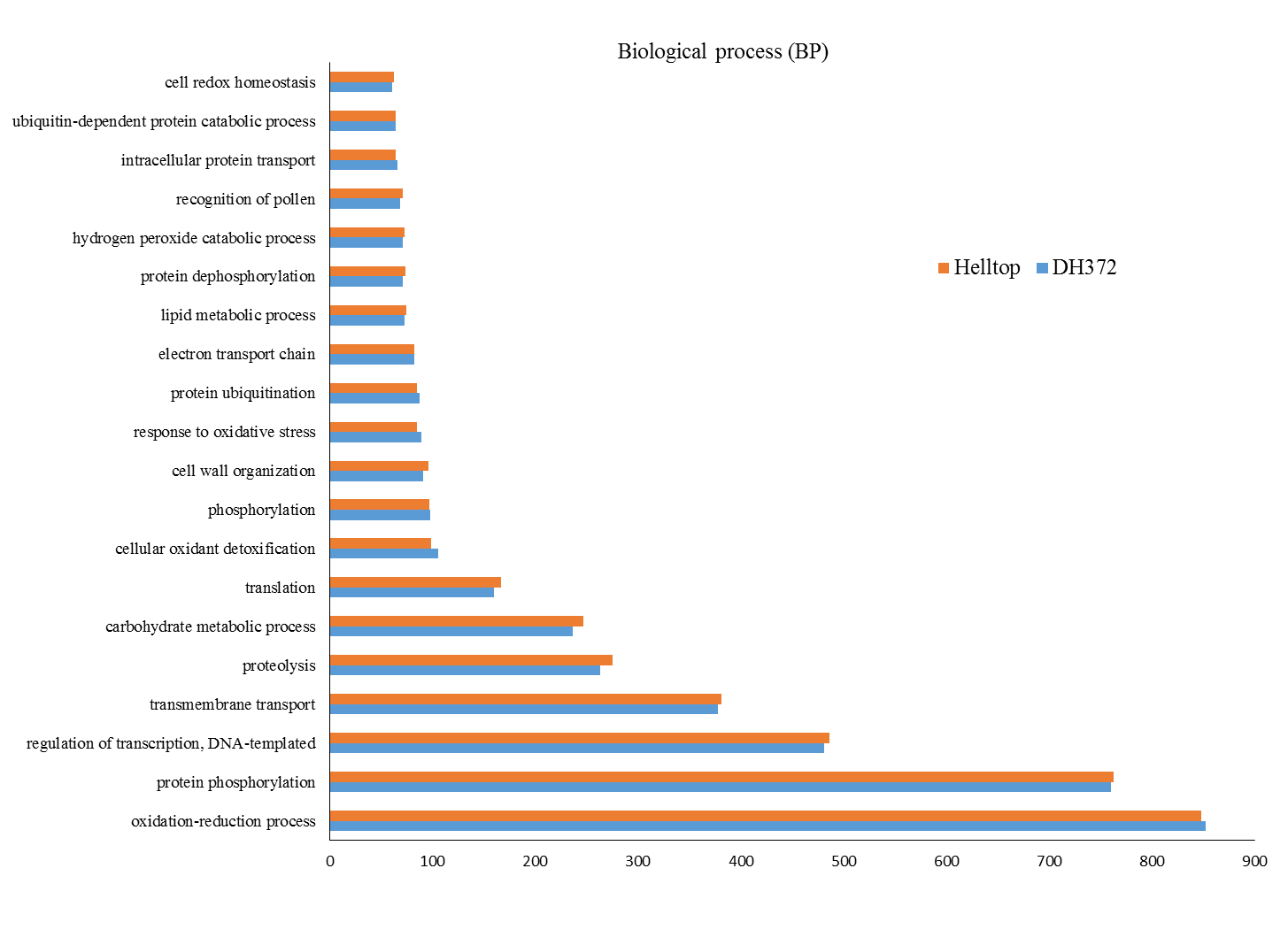

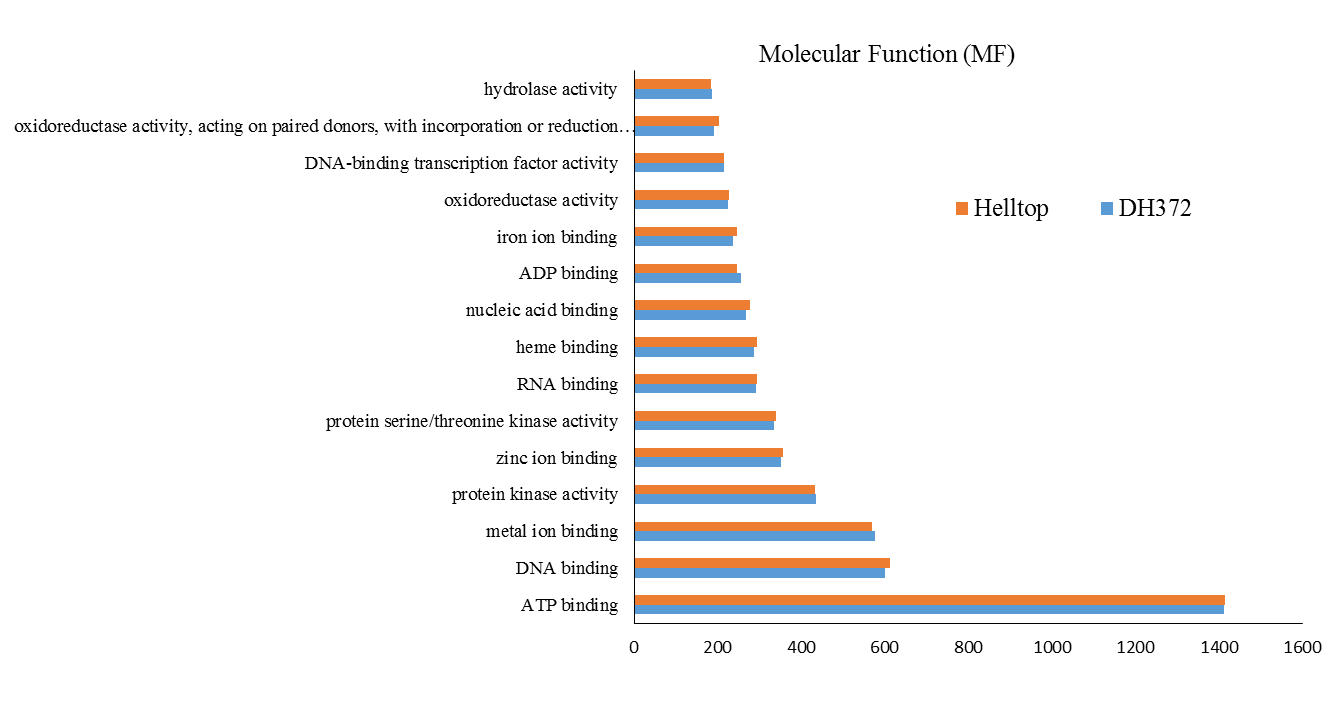

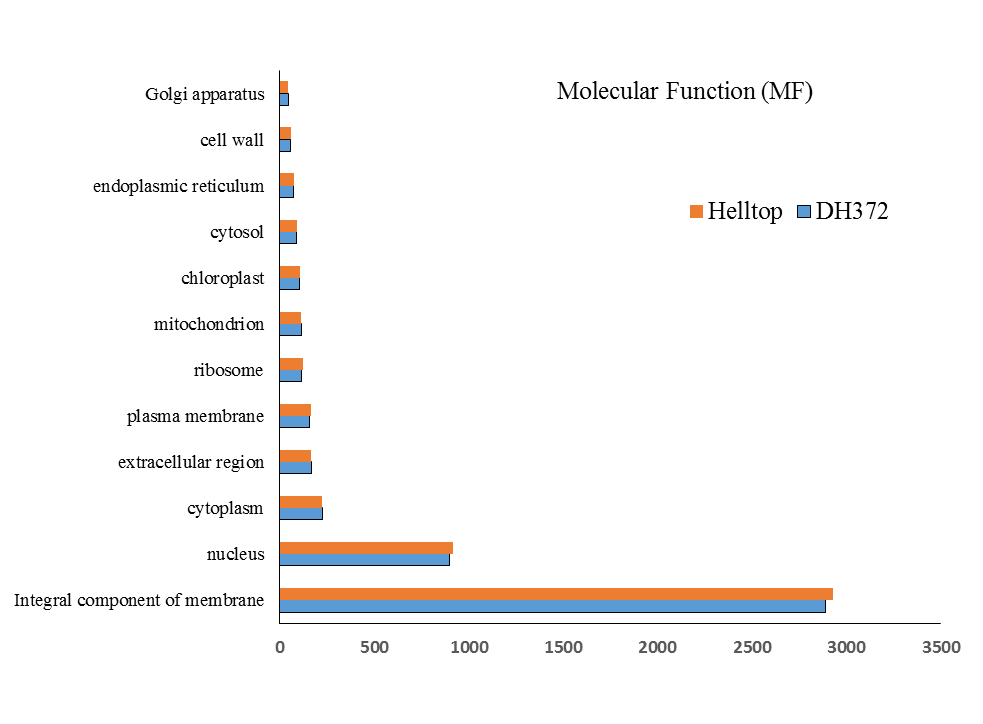


**A**

**B**

**C**

**Supplementary Fig 2: Distribution of Gene ontology terms in the assemblies of DH372 and Helltop.** A: biological process. B: Cellular component. C: Molecular functi

**Supplementary Table 1: Top 30 differentially expressed genes ergot specific genes**

| Tags | Name | Description | Length bp | FC | logFC | logCPM | P-Value |
| --- | --- | --- | --- | --- | --- | --- | --- |
| [UP] | XLOC_1382173 | hypothetical protein TRIUR3_06896 | 690 | 5410 | 12.40152 | 5.682046 | 2.36E-05 |
| [UP] | XLOC_1367953 | unnamed protein product | 5381 | 3716 | 11.8597 | 10.29918 | 5.79E-06 |
| [UP] | XLOC_150571 | unnamed protein product | 4040 | 3613 | 11.81894 | 7.097602 | 1.01E-05 |
| [UP] | XLOC_293296 | aldehyde oxidase GLOX-like | 3505 | 2367 | 11.20914 | 7.182831 | 5.17E-05 |
| [UP] | XLOC_1018663 | proline-rich receptor-like protein kinase PERK8 | 4656 | 2310 | 11.17355 | 6.417845 | 4.98E-06 |
| [UP] | XLOC_864564 | Actin-depolymerizing factor 6 | 1157 | 2167 | 11.0812 | 6.903354 | 0.000188 |
| [UP] | XLOC_120399 | predicted protein | 3465 | 2094 | 11.03183 | 6.662731 | 3.07E-05 |
| [UP] | XLOC_145626 | unnamed protein product | 4013 | 1784 | 10.80051 | 5.830042 | 5.31E-05 |
| [UP] | **XLOC_1432429** | **COBRA-like protein 10** | 2465 | 1594 | 10.63887 | 7.34373 | 1.41E-05 |
| [UP] | XLOC_1185420 | uncharacterized protein LOC109787628 | 751 | 1518 | 10.56804 | 5.885757 | 1.55E-05 |
| [UP] | XLOC_223785 | berberine bridge enzyme-like 18 | 2787 | 1492 | 10.5433 | 4.530702 | 8.04E-06 |
| [UP] | **XLOC_118963** | **probable pectinesterase/pectinesterase inhibitor 21** | 2829 | 1346 | 10.39492 | 7.785668 | 4.97E-05 |
| [UP] | XLOC_109980 | pollen-specific leucine-rich repeat extensin-like protein 3 isoform X1 | 5661 | 1230 | 10.26399 | 8.625102 | 9.54E-05 |
| [UP] | XLOC_201604 | unnamed protein product | 597 | 1217 | 10.24962 | 6.273857 | 0.000129 |
| [UP] | XLOC_144374 | ---NA--- | 627 | 1193 | 10.22058 | 3.772508 | 0.000102 |
| [DOWN] | XLOC_250266 | unnamed protein product | 2180 | -117 | -6.8749 | 1.680019 | 4.58E-08 |
| [DOWN] | XLOC_383740 | vacuolar-sorting receptor 1-like isoform X1 | 3310 | -126 | -6.97339 | 2.00972 | 2.67E-06 |
| [DOWN] | XLOC_1202202 | predicted protein | 2199 | -212 | -7.72926 | 2.898601 | 5.87E-08 |
| [DOWN] | XLOC_836618 | ---NA--- | 516 | -222 | -7.79625 | 2.068088 | 2.81E-06 |
| [DOWN] | XLOC_1337707 | putative disease resistance protein RGA4 | 6720 | -276 | -8.11076 | 2.611713 | 2.98E-07 |
| [DOWN] | XLOC_1043319 | unnamed protein product | 252 | -335 | -8.38857 | 1.807848 | 1.48E-06 |
| [DOWN] | XLOC_225997 | monomeric alpha-amylase inhibitor | 456 | -359 | -8.48608 | 1.937677 | 1.07E-07 |
| [DOWN] | XLOC_221444 | putative glutathione S-transferase GSTF1 | 1724 | -366 | -8.51497 | 2.092008 | 0.000651 |
| [DOWN] | XLOC_187705 | unnamed protein product | 1831 | -466 | -8.86329 | 3.313807 | 2.17E-11 |
| [DOWN] | XLOC_413575 | uncharacterized protein LOC112895447 | 384 | -485 | -8.92052 | 3.028695 | 9.49E-08 |
| [DOWN] | XLOC_047910 | uncharacterized protein LOC109734041 | 3061 | -583 | -9.18683 | 5.167587 | 1.24E-21 |
| [DOWN] | XLOC_042367 | unnamed protein product | 1293 | -594 | -9.21451 | 3.504222 | 1.13E-07 |
| [DOWN] | XLOC_1031999 | probable glutathione S-transferase BZ2 | 726 | -771 | -9.58996 | 3.142837 | 1.68E-05 |
| [DOWN] | XLOC_064721 | retrotransposon protein. putative. unclassified | 1527 | -1162 | -10.1829 | 4.365998 | 1.1E-14 |
| [DOWN] | XLOC_1263374 | thaumatin-like protein TLP3 | 1004 | -3675 | -11.8437 | 5.048639 | 0.00011 |

**^FC= Fold change, CPM= count per million, Genes highlighted in the bold are also identified in enriched pathway cell wall modification^**


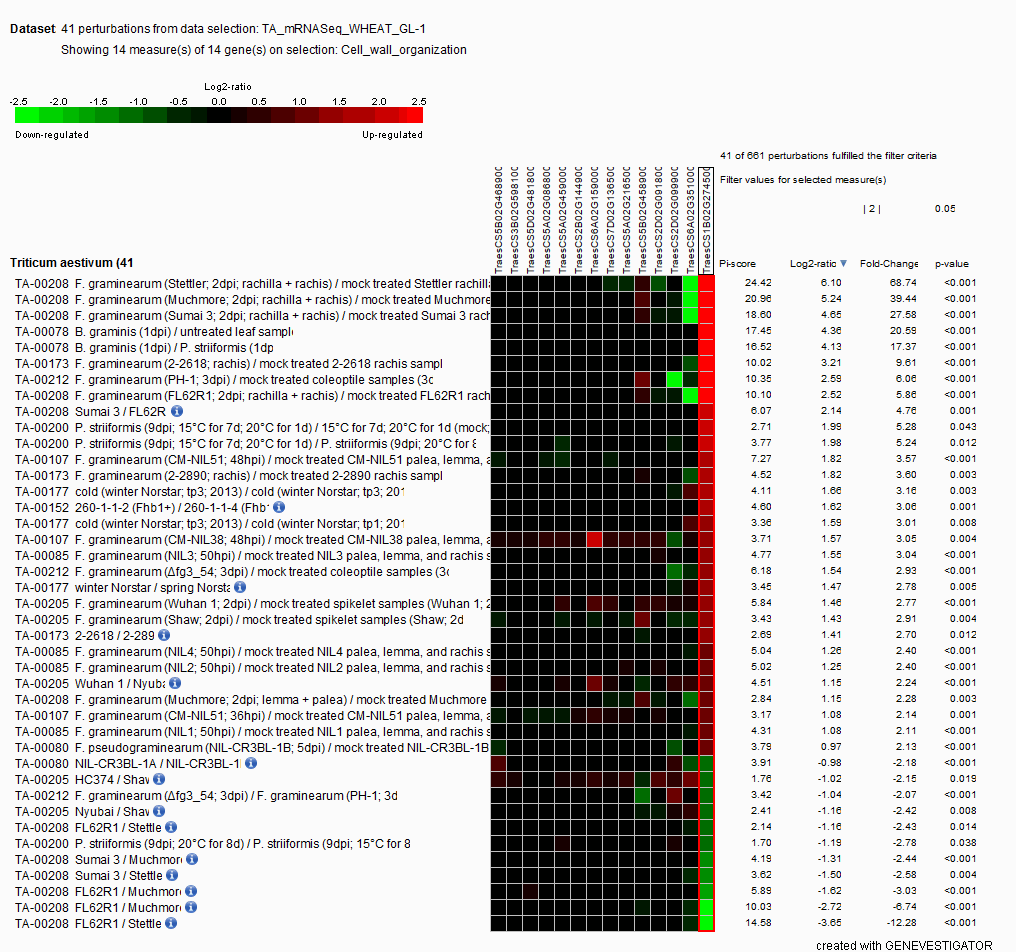

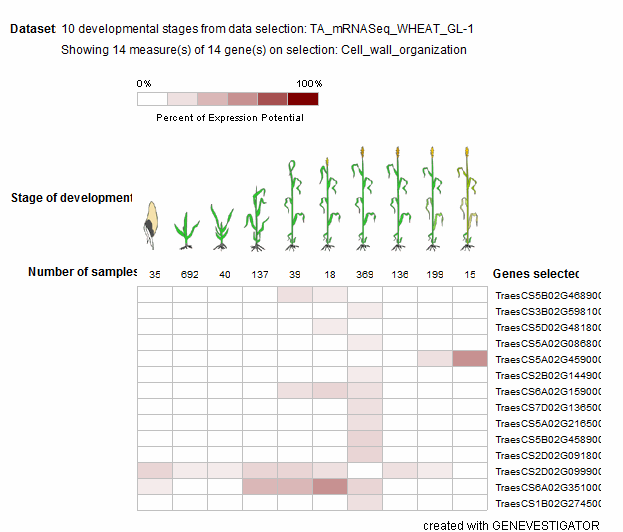


**A**

**B**

**Supplementary Fig 3:** **Gene expression pattern wheat orthologs of pectinestrase and cell wall modification genes** (A) Developmental stage-specific expression pattern B) Heat map of expression of selected genes in response to various external conditions such as biotic stress were analyzed using Genevestigator perturbation tool.
